# The Army Dental Examining Board

**Published:** 1901-09

**Authors:** K. J. Schumann

**Affiliations:** Athens, Tenn.


### Domestic Correspondence.

### THE ARMY DENTAL EXAMINING BOARD.

To the Editor:

Sir,—The general impression among certain members of the dental profession regarding the personnel of the Army Examining Board is far from a favorable one. The journals over the country have all had something to remark regarding this board, its proficiency in a professional way, and its competency as an examining board. One of these very prominent journals went so far as to say that there was one well-known man on this board, insinuating that perhaps there was only one competent man there.

That which I have to write regarding this board is purely the promptings of gratitude and with the hope of correcting some erroneous impressions. While I realize that the gentlemen comprising this board do not desire any public mention in this direction, I trust that my remarks will not be regarded as ill-timed. It is known that quite a number of applicants have gone before this board and failed to pass what was then termed an unfair examination. Some of these have informed me that the subjects dealt with were obscure and the questions unfair. I wish to give my testimony in this matter, and that I have been before this board and virtually failed to pass, or rather was compelled to withdraw on account of sickness. I can clearly remember the kindness with which the members dealt with an applicant. They do not occupy an enviable position by any means, and while they do not offer to teach a candidate, as some seem to think they should, they still use every fair and honest means available to bring out what man has in him. The one characteristic feature of this board is its profound respect for a professional gentleman. One is treated simply as an equal; and though some of the candidates fail to appreciate this fact, and take advantage of the courtesies shown them, the members of the board never allow one to lose sight of the fact that they are doing their duty and are not there for the purpose of frightening a man out of what he knows. The examination is rigid, and it certainly should be so, but as to the questions being unfair or obscure, there is positively no such thing existing.

Among a select few who have failed this report of unfairness and incompetence has arisen, and some of these individuals are sure to meet a new candidate and give him false impressions regarding the examination and the men who are holding it.

I believe it is due the board that these reports be met, at least, with the truth from some source. These gentlemen have paid little or no attention to these unkind rumors, and some of them are very unkind indeed, being of a purely personal nature and questioning the honesty and competency of them as a board.

The general impression that political pull is all that is necessary to enable one to pass is certainly a most unjust assertion, as I know that it avails but little. As a whole, I never saw a more perfect organization than this board of three. I never saw gentlemen so harmoniously laboring, and I believe that they give a candidate credit for everything he knows, and I certainly know that the marks are generous. They take nothing for granted.

In conclusion, I desire to say that personally the Army Board of Dental Examiners has my lasting gratitude for the courtesy extended me, and as a board I pay tribute to their fairness, honesty, competency, and professional dealing with profesional gentlemen.

K. J. SCHUMANN.

ATHENS, TENN.

